# Comparative Thermal and Fire Behavior of Rigid Polyurethane (PUR) and Polyisocyanurate (PIR) Foams Formulated with Recycled Poly(ethylene terephthalate) (PET) Polyols—Part 1

**DOI:** 10.3390/ma19030525

**Published:** 2026-01-28

**Authors:** Mateusz Skowron, Urszula Lelek-Borkowska, Karolina Kaczmarska

**Affiliations:** Faculty of Foundry Engineering, AGH University of Krakow, Reymonta 23 St., 30-059 Krakow, Poland; lelek@agh.edu.pl (U.L.-B.); karolina.kaczmarska@agh.edu.pl (K.K.)

**Keywords:** polyurethane (PUR), polyisocyanurate (PIR), thermal insulation, thermal conductivity, fire properties, fire behavior, flame spread, ignition, recycled PET, polyester polyol

## Abstract

**Highlights:**

**What are the main findings?**

- PIR foams with recycled PET-based polyester polyols show delayed thermal degradation and a char yield increase from ~3 wt.% to >22 wt.%.
- The PIR system exhibits a higher glass transition temperature and improved thermo-oxidative stability compared with PUR foam.
- Peak heat release rate is reduced by ~50% in PIR foams, indicating significantly lower fire intensity.

**What are the implications of the main findings?**

- PIR foams demonstrate slower fire growth and enhanced condensed-phase stabilization during combustion.
- Halogen-free PIR formulations produce lower toxic gas emissions than conventional PUR systems.
- Recycled PET-derived polyols improve fire performance while supporting sustainable material design for advanced insulation applications.

**Abstract:**

Rigid polyurethane (PUR) and polyisocyanurate (PIR) foams are widely used as thermal insulation materials due to their excellent thermal conductivity and low density. However, fire resistance remains a critical property determining their safe application in construction, transportation, and energy systems. This study provides a comparative overview of the fire behavior of PUR and PIR foams, focusing on structural aspects, decomposition mechanisms, flame retardancy, and performance of emission of toxic gases during the combustion process. Despite extensive studies on PUR and PIR foams, systematic comparative investigations addressing the combined influence of recycled PET-based polyester polyols, isocyanurate content, and fire-related properties—including thermal degradation, heat release, and toxic gas emissions—remain limited. PIR foams, characterized by higher isocyanate indices and the presence of isocyanurate rings, show superior thermal stability, reduced heat release rates, and enhanced char formation compared with PUR foams. Experimental analysis of thermal degradation (TGA/DTG) and heat release (cone calorimetry) confirms that PIR foams demonstrate higher resistance to ignition and slower fire propagation. The results emphasize the critical role of molecular architecture and crosslink density in shaping the fire performance of rigid foams, highlighting PIR systems as advanced insulation solutions for applications requiring stringent fire safety standards. The PIR foam was prepared using a polyester polyol derived from recycled PET, which could help in achieving better fire properties during the combustion process. Compared with PUR foams, PIR foams exhibited an approximately 50% reduction in peak heat release rate, an increase in char yield from about 3 wt.% to over 22 wt.%, and a shift of the main thermal degradation peak by approximately 55 °C toward higher temperatures, indicating substantially enhanced fire resistance.

## 1. Introduction

Rigid polyurethane (PUR) and polyisocyanurate (PIR) foams are among the most widely used thermal insulation materials in the construction, transportation, and refrigeration industries. While both materials offer very low thermal conductivity, excellent strength-to-weight ratio, and good processing versatility, their long-term durability and fire resistance differ significantly due to their distinct chemical architectures. In industrial practice, these differences are not only a function of chemical formulation but also of processing parameters such as isocyanate index, catalyst balance, blowing agent selection, and cure kinetics—all of which influence the resulting polymer network topology [1,2,3,4,5,6].

The fire performance of polymeric foams is intimately linked to their thermal decomposition mechanisms, which govern ignition behavior, flame spread, production of combustible volatiles, and char formation efficiency. Among the available analytical tools, thermogravimetric analysis (TG) combined with derivative thermogravimetry (DTG) represents one of the most fundamental and quantitative methods to investigate polymer stability and degradation kinetics. TG allows for the precise determination of the onset of decomposition, the number of distinct degradation steps, and the residual char yield under both inert and oxidative atmospheres. The corresponding DTG curves enhance interpretative resolution by enabling the separation of overlapping degradation events and the assignment of mass-loss steps to specific chemical transformations within the polymer matrix [7,8,9].

In the case of conventional PUR foams, degradation typically proceeds through two dominant events. The first involves the cleavage of urethane bonds at approximately 200–280 °C, a process associated with the formation of primary isocyanates, secondary amines, and several volatile oligomeric fragments. This stage is strongly influenced by the nature of the polyol (polyether vs. polyester), the degree of crosslinking, and the presence of catalysts such as tertiary amines or organometallic compounds that can accelerate bond scission. The second degradation event occurs within the 320–380 °C range and corresponds to the breakdown of the polyol-rich soft segments and residual crosslinks. This stage is often accompanied by the release of combustible gases including CO, CO_2_, alkenes, and various carbonyl-containing species, which collectively promote flame propagation and contribute to higher peak heat release rates observed during fire testing [7,8,9,10].

By contrast, PIR foams, synthesized under elevated isocyanate indices (typically 250–350), develop a network rich in isocyanurate rings generated through the cyclotrimerization of isocyanate groups. These triazine-like structures exhibit substantially higher thermal stability, with bond dissociation energies exceeding those of urethane linkages by nearly a factor of two. As a result, the primary decomposition of PIR materials is delayed until ~350–400 °C, with the DTG curve often showing a much broader and less intense peak relative to PUR. Importantly, the condensed-phase degradation of the isocyanurate network yields a significantly larger proportion of carbonaceous char—a rigid, thermally insulating layer that suppresses mass transport, retards further degradation, and reduces heat release during fire exposure [11,12].

In addition to the inherent stability of the isocyanurate structures, several formulation and processing parameters further modulate the decomposition characteristics of PIR foams. Pentane and HFO blowing agents influence initial foam morphology and closed-cell content, which affects oxygen diffusion and thermal gradient formation during heating [8,13,14]. Catalysts such as potassium octoate enhance trimerization, increasing crosslink density and promoting more cohesive char formation. Moreover, high index PIR formulations often incorporate phosphorus-based flame retardants, which contribute to additional condensed-phase stabilization via the formation of phosphoric and polyphosphoric acid species during thermal stress [15,16]. For the foams produced in this study, halogen-free and phosphorus-free flame retardants were used. As a result, the material generates significantly lower amounts of harmful substances during combustion, minimizing the release of toxic gases and environmentally persistent compounds into the atmosphere [17,18].

This study also places strong emphasis on the use of recycled materials, which has become a central principle in modern material design within the polyurethane engineering industry. The use of PET-based polyester polyols is particularly important today due to increasing environmental pressures, the need to reduce plastic waste, and the global shift toward circular-economy manufacturing [19]. Incorporating recycled PET into polyurethane and polyisocyanurate foam formulations not only diverts significant amounts of post-consumer plastic from landfills and incineration, but also lowers the carbon footprint of the final product. Moreover, PET-derived polyols often exhibit favorable mechanical and thermal characteristics, enabling manufacturers to achieve high-performance insulation materials while simultaneously meeting sustainability targets and regulatory requirements. Due to the chemical structure of PET, materials produced using PET-based polyester polyols exhibit higher thermal stability compared with conventional polyurethane materials [20,21,22,23].

Thus, TGA/DTG not only quantifies the differences in decomposition behavior between PUR and PIR foams but also provides mechanistic insight into how molecular architecture, crosslink density, foam morphology, and the presence of flame-retardant additives influence global thermal stability [22,24]. For these reasons, the present study places particular emphasis on comparative thermal analysis to illustrate the superior thermo-oxidative resistance and fire performance of PIR foams relative to PUR foams, reinforcing the critical importance of structural design in polymeric insulation materials. Typical polyurethane foam properties have been shown in Table 1 [14,25].

Although the fire performance of conventional PUR and PIR foams has been extensively investigated—covering aspects such as thermal stability, flammability, and flame-retardancy strategies—rigid polymeric foams remain intrinsically flammable and prone to rapid heat release and toxic gas evolution during combustion [6,9,26,27].

In recent years, increasing attention has been devoted to sustainable polyurethane systems prepared from waste polymers, including recycled polyester polyols derived from poly(ethylene terephthalate) (PET) [6]. However, most of these studies have primarily focused on synthesis routes, mechanical performance, and thermal insulation properties, while integrated analyses of fire behavior and toxic gas emissions remain limited. Moreover, available literature typically investigates PUR and PIR foams separately and under varied formulation and testing conditions, which hampers direct comparison of their thermal degradation pathways and combustion responses.

The literature confirms that PIR foams generally exhibit improved fire performance compared with PUR foams due to the presence of highly crosslinked isocyanurate networks and enhanced condensed-phase stabilization during combustion [26,28,29]. At the same time, the influence of recycled PET-based aromatic polyester polyols on the fire behavior of PIR systems remains insufficiently addressed, and direct comparative studies of PUR and PIR foams formulated with the same family of recycled PET-based polyols and evaluated under identical conditions are still scarce [5,30,31,32].

Therefore, the objective of this study is to systematically compare the thermal stability, fire behavior, and toxic gas emissions of PUR and PIR foams incorporating recycled PET-based polyester polyols using a consistent formulation strategy and an integrated experimental approach.

## 2. Methodology

The research program consisted of (i) formulation and preparation of comparable PUR and PIR foams with controlled density, (ii) thermal characterization by TG–DTG and DSC, (iii) evaluation of fire performance using cone calorimetry, and (iv) assessment of toxic gas emissions according to PN-B-02855. Comparative analysis was based on degradation onset temperatures, char yield, glass transition temperature, heat release parameters, and toxicometric indices.

### 2.1. Materials

The following chemical components were used in this study to prepare the polyurethane (PUR) and polyisocyanurate (PIR) foam systems. All raw materials were employed as received, without further purification:

- Rokopol^®^ G44—a polyether polyol with a viscosity of 2500–3100 mPa·s (supplied by PCC Rokita, Brzeg Dolny, Poland). This polyol served as the primary polyether component for the PUR formulations.
- TERATE^®^ HT 2000—a recycled PET-based polyester polyol with a viscosity of 5000–8000 mPa·s (supplied by Stepan Company, Maywood, NJ, USA). This component was used particularly in PIR formulations to enhance thermal stability and char formation due to its aromatic polyester structure.
- Voracor™ CD 345—polymeric diphenylmethane diisocyanate (pMDI; supplied by Dow Chemicals, Midland, MI, USA). This isocyanate was used as the reactive component in both PUR and PIR systems, with varying isocyanate indices depending on the foam type.
- n-Pentane—a physical blowing agent (boiling point 36 °C) supplied by ProChema GmbH, Wien, Austria. The blowing agent was used to generate the cellular structure in both foam types.
- Amine catalysts—PMDETA (20% solution) and Polycat^®^ 5, both supplied by Evonik Industries AG, Essen, Germany). These tertiary amine catalysts were used to promote urethane formation and regulate the balance between gelling and blowing reactions.
- DABCO^®^ TMR-2—a quaternary ammonium salt dissolved in glycol (Evonik Industries AG, Essen, Germany). This catalyst selectively promotes the isocyanurate trimerization reaction and was therefore essential for PIR foam synthesis.
- Potassium octoate—a standard metal–organic catalyst widely used in rigid PUR/PIR formulations to support both urethane and isocyanurate reaction pathways (Milliken & Company, Spartanburg, SC, USA).
- Triethyl phosphate (TEP)—a liquid, halogen-free fire retardant (ProChema GmbH, Wien, Austria). TEP was incorporated into selected formulations to enhance the flame-retardant performance without introducing halogenated species, ensuring lower toxicity of combustion products.

### 2.2. Formulation Design

All components were stored and handled under controlled laboratory conditions (21 °C, 24 h) to ensure proper reactivity and consistency during foam production.

Two groups of formulations were developed for the purpose of this study: polyurethane (PUR) foams produced at a low isocyanate index and polyisocyanurate (PIR) foams synthesized under high-index conditions. The design of each formulation was based on the functional roles of the polyols, catalysts, fire retardants, and blowing agents, as well as the targeted cellular structure and thermal performance.

For the PUR foams, Rokopol^®^ G44 served as the primary polyether polyol. The formulation was designed to achieve a balanced reactivity profile, ensuring proper synchronization between the gelling and blowing reactions. PMDETA and Polycat^®^ 5 were used as the main amine catalysts to promote urethane formation. A polymeric MDI (Voracor™ CD 345) was used at an index of 100–110 to achieve a flexible yet sufficiently crosslinked network. n-Pentane was incorporated as the physical blowing agent to generate a fine and uniform cellular structure. Triethyl phosphate (TEP) was included as a halogen-free flame retardant to improve combustion behavior without introducing toxic halogenated species.

In contrast, the PIR foams were formulated with a significantly higher isocyanate index (typically 250–300) to promote extensive cyclotrimerization of the isocyanate groups. The PET-based polyester polyol TERATE^®^ HT 2000 was introduced to enhance aromaticity, thermal stability, and char-forming capability. DABCO^®^ TMR-2, a quaternary ammonium salt catalyst, was used to accelerate isocyanurate ring formation, while potassium octoate served as a co-catalyst to support both urethane and trimerization pathways. n-Pentane was again used as the blowing agent, and TEP was added to further improve flame resistance through condensed-phase action. The formulation strategy intentionally leveraged the synergistic effect between isocyanurate network formation and the aromatic polyester structure to obtain a material with superior fire performance.

The overall formulation design ensured that both foam systems—PUR and PIR—were comparable in terms of target density (approximately 35–40 kg/m^3^), while exhibiting distinct differences in chemical architecture, crosslink density, and expected thermal decomposition behavior. These differences allowed for a direct structure–property comparison in subsequent analyses.

### 2.3. Foam Preparation

All foam samples were prepared using a high-speed laboratory mixer (Ika Eurostar 20, IKA-Werke GmbH & Co. KG, Staufen im Breisgau, Germany) to ensure reproducible mixing conditions and homogeneous dispersion of all formulation components. Prior to processing, all raw materials (polyols, catalysts, blowing agents, fire retardants, and isocyanate) were equilibrated to room temperature (22 ± 1 °C) to maintain consistent reactivity and viscosity.

For each formulation, the polyol blend was prepared by combining the required amounts of polyols, catalysts, blowing agent (n-pentane), TEP, and any additional additives in a stainless-steel beaker (Table 2 and Table 3). The polyol-side components were premixed for 30–60 s at 2000–2500 rpm, ensuring full homogenization, proper emulsification of the blowing agent, and uniform distribution of catalysts. Care was taken to minimize the loss of pentane during premixing by covering the vessel immediately after addition.

After homogenization of the polyol component, the calculated amount of polymeric MDI (Voracor™ CD 345) was added to the mixture. The isocyanate and polyol components were mixed for 6–8 s at 3000–3500 rpm, depending on the reactivity of the formulation (lower for PUR, higher for PIR systems). The mixing time was optimized to achieve proper nucleation and consistent cream times without inducing excessive mechanical frothing.

Immediately after mixing, the reacting mixture was poured into an open mold (300 mm × 300 mm× 50 mm) and allowed to freely rise under atmospheric pressure. The foams were cured at ambient conditions for 24 h (temp. 21 °C, humidity 33.5%) after which they were demolded and conditioned for an additional 72 h prior to testing (temp. 21 °C, humidity 33.5%). For PIR formulations, extended post-curing was applied when necessary to ensure full development of the isocyanurate network.

**Figure 1:**
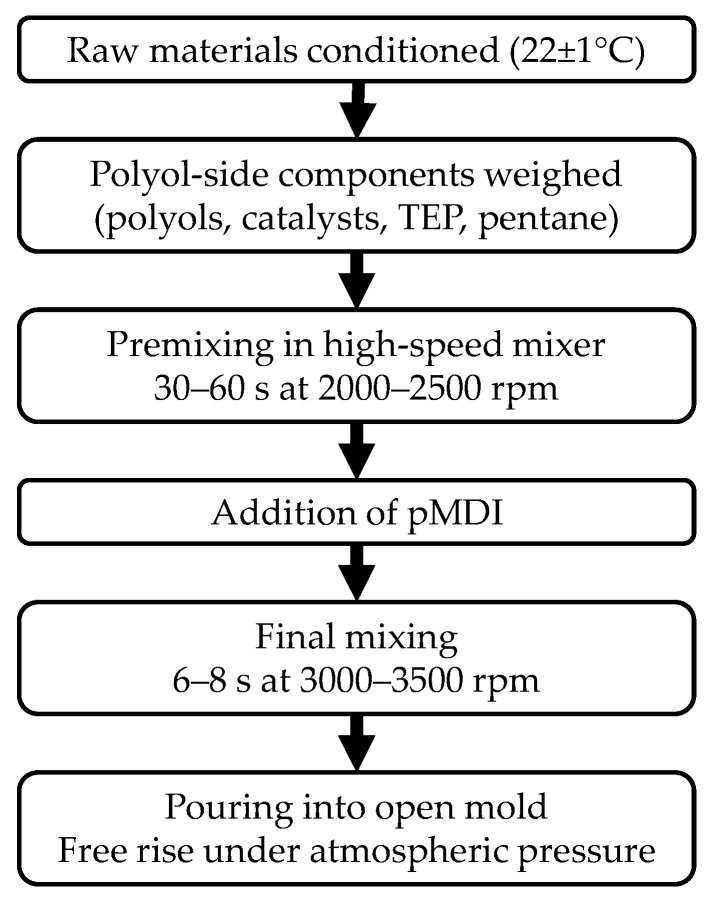
Foam preparation pathway.

Throughout the preparation process, the mixer speed, component temperature, and mixing times were kept constant to ensure comparability across PUR and PIR foam systems. All samples were then cut into standardized dimensions according to the respective testing protocols. Overall the process has been shown on Figure 1.

### 2.4. Thermal Analysis by TG-DTG-DSC

The thermogravimetric analysis (TG–DTG) of the PUR and PIR foam samples was performed in accordance with PN-EN ISO 11358-1:2014-09 [33] using a Mettler-Toledo TGA/SDTA 851e thermogravimetric analyzer (Mettler-Toledo International Inc., Columbus, OH, USA). The measurements were conducted over a temperature range of 25–900 °C at a controlled heating rate of 10 °C/min. All tests were carried out under a synthetic air atmosphere with a purity of 99.9992%, and the gas flow rate through the furnace was set to 60 mL/min, ensuring stable oxidative degradation conditions. The samples were placed in Al_2_O_3_ crucibles, which provided high thermal and chemical stability during testing.

Differential scanning calorimetry (DSC) was performed in accordance with PN-EN ISO 11357-1:2016-11 [34], PN-EN ISO 11357-2:2020-09 [35], and PN-EN ISO 11357-3:2018-06 [36] using a Mettler-Toledo DSC 822e calorimeter (Mettler-Toledo International Inc., Columbus, OH, USA). The main objective of the DSC analysis was to characterize the thermal transitions of the foam systems, including glass transition temperature (*T_g_*), post-cure enthalpy changes, and soft-segment mobility. The tests were carried out over a temperature range of 0–180 °C, using a heating/cooling rate of 10 °C/min, with 5 min isothermal segments at the start and end of each cycle.

For PIR samples, which exhibit higher thermal stability due to the presence of isocyanurate structures, an extended temperature range of 0–210 °C was applied to ensure full capture of all relevant transitions. Measurements were conducted in a synthetic air atmosphere (99.9992% purity) at a constant flow of 60 mL/min. The samples were placed in 40 μL high-purity aluminum crucibles with perforated lids, which provided controlled venting and minimized pressure buildup during decomposition events. The initial mass of DSC samples ranged between 8 and 12 mg, depending on foam type and density.

### 2.5. Cone Calorimetry HRR Analysis

Cone calorimetry measurements were performed in accordance with ISO 5660-1:2015 [37] using a dual-cone fire testing system (Fire Testing Technology Ltd., East Grinstead, UK). The apparatus (Netzsch TCC918 by NETZSCH-Gerätebau GmbH, Selb, Germany) is equipped with a controlled radiant heat source, precision load cell, spark ignition module, exhaust gas analysis system (O_2_, CO, CO_2_), and an integrated system for calculating heat release based on oxygen consumption calorimetry.

Test conditions:

- external heat flux: 35 kW·m^−2^ (standard exposure for evaluating polymeric insulation foams)
- specimen dimensions: 100 mm × 100 mm × 25 mm
- sample orientation: horizontal, exposed surface facing the cone heater
- ignition method: continuous pilot spark ignition (10 kV)
- exhaust flow rate: 24 ± 2 L·s^−1^, calibrated before each test
- environmental conditions: temperature: 23 ± 2 °C, relative humidity: 50 ± 5%.

### 2.6. Toxic Gas Emission Analysis

The assessment of toxic gaseous products released during thermal decomposition and combustion of the PIR foams was performed at Laboratory of Material Flammability Testing. The methodology followed the requirements of the Polish standard PN-B-02855 Fire protection of buildings—Testing method of emission of toxic products of decomposition and combustion of materials [38].

The toxicometric index WLC50 and its modified form WLC50M were determined according to PN-B-02855. These indices quantify the concentration of toxic gases that would cause 50% lethality in standardized laboratory conditions. The following toxic gases were monitored in the combustion chamber:

- carbon monoxide (CO)
- carbon dioxide (CO_2_)
- hydrogen cyanide (HCN)
- nitrogen dioxide (NO_2_)
- sulfur dioxide (SO_2_).

Measurements were performed at three controlled decomposition temperature values: 450 °C, 550 °C, and 750 °C, reflecting early-stage heating, advanced decomposition, and high-temperature oxidative breakdown of PIR structures.

## 3. Results and Discussion

### 3.1. Thermal Analysis (TG-DTG-DSC)

**Figure 2:**
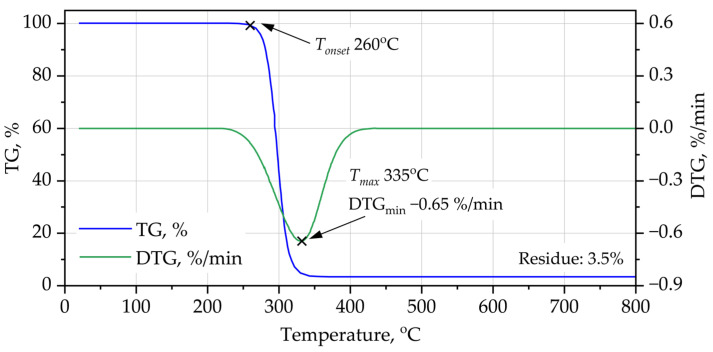
TG-DTG curves of PUR foam.

**Figure 3:**
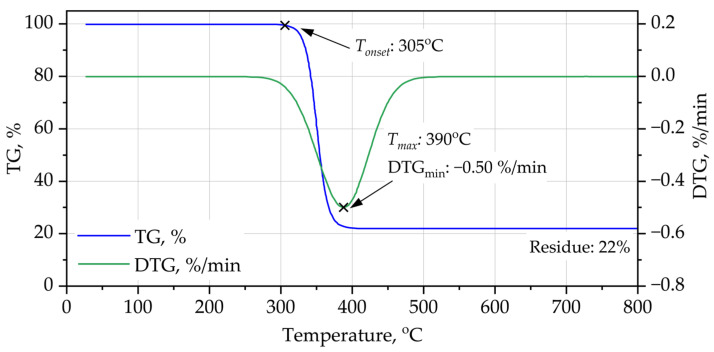
TG-DTG curves of PIR foam.

The thermal stability and degradation behavior of the PUR and PIR foams were evaluated using TG–DTG shown on Figure 2 and Figure 3 under oxidative conditions. The results clearly demonstrate substantial differences in the onset of degradation, the maximum decomposition rates, and the char yield, reflecting the influence of chemical architecture on thermal resistance.

The PUR foam undergoes two-step degradation typical of polyether-based polyurethane systems. The first significant mass-loss event begins at approximately 260 °C, corresponding to urethane bond dissociation and volatilization of isocyanate- and polyol-derived fragments. The DTG peak indicates a maximum degradation rate at around 335 °C, followed by nearly complete volatilization of the organic matrix, leaving only ~3.5 wt.% residual char at 800 °C. This low char yield reflects the limited aromatic content and the absence of thermally stable ring structures in the PUR network.

In contrast, the PIR foam shows markedly enhanced thermal resistance. The onset of major mass loss is shifted to ~305 °C, and the DTG peak occurs at a significantly higher temperature (~390 °C). More importantly, PIR exhibits a substantially higher char yield (~22 wt.% at 800 °C), indicative of the thermally robust isocyanurate rings formed at elevated isocyanate index. The higher char content is consistent with the triazine-like structure of isocyanurate groups and the use of PET-based polyester polyol, both of which promote carbonaceous char formation during oxidative degradation. The delayed onset and slower degradation kinetics explain the superior fire resistance observed in cone calorimetry (lower peak HRR and longer time to peak). Overall, the TG–DTG curves confirm that PIR foam is significantly more thermally stable than PUR foam, primarily due to its higher degree of aromaticity, crosslink density, and thermally stable isocyanurate backbone.

The enhanced thermal stability of PIR foams cannot be attributed solely to the presence of isocyanurate rings or aromatic polyester segments individually, but rather to their synergistic interaction during thermal degradation. The isocyanurate network delays the initial bond scission by providing a highly crosslinked and thermally stable backbone, while the aromatic PET-derived polyester segments promote early-stage carbonization and char cohesion. As a result, volatile degradation products are released more gradually, and a continuous carbonaceous structure is formed in the condensed phase, effectively slowing down mass loss and shifting the maximum degradation rate toward higher temperatures.

**Figure 4:**
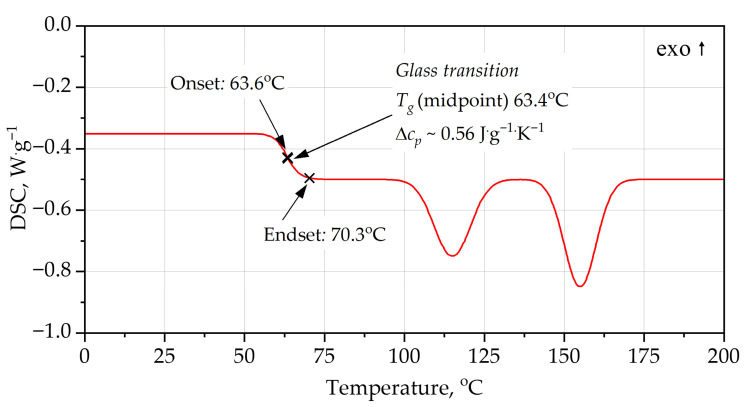
DSC curves of PUR foam, heating.

**Figure 5:**
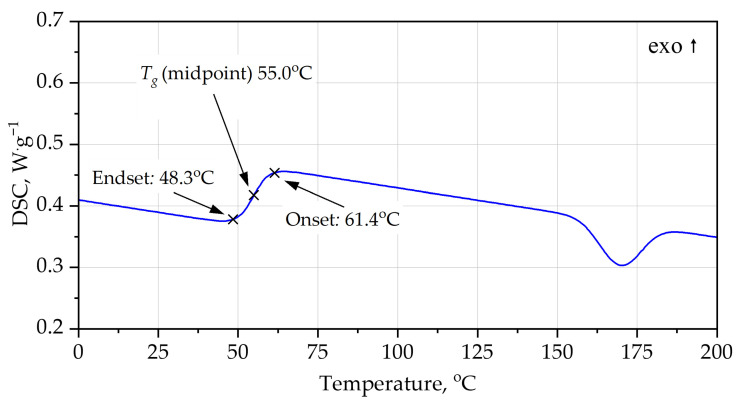
DSC curves of PUR foam, cooling.

**Figure 6:**
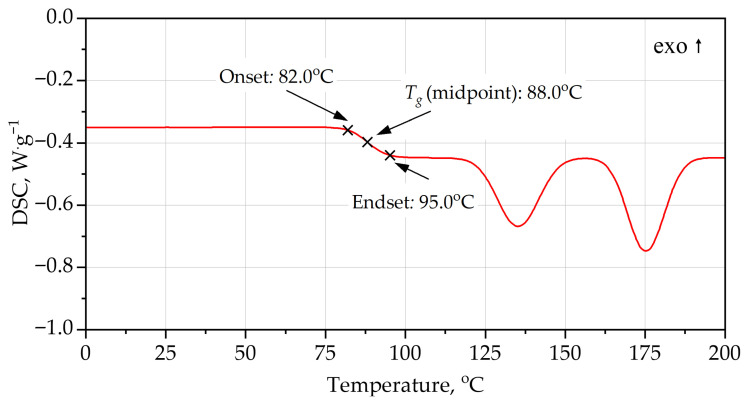
DSC curves of PIR foam, heating.

**Figure 7:**
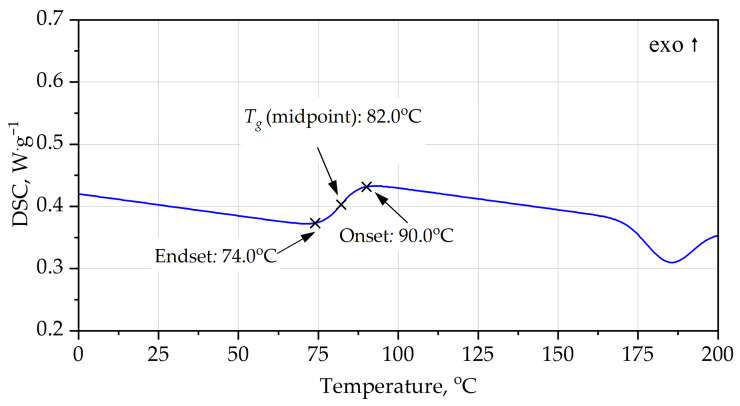
DSC curves of PIR foam, cooling.

DSC analysis Figure 4, Figure 5, Figure 6 and Figure 7 provides additional insight into the segmental mobility and structural organization of the foams. For PUR, the heating curve reveals a well-defined glass transition at *T_g_* ≈ 63–64 °C, with Δ*c_p_* ≈ 0.56 J·g^−1^·K^−1^. The transition is relatively sharp, reflecting microphase-separated soft segments typical of polyether-based PUR systems. During cooling, *T_g_* shifts to a lower temperature (midpoint ~55 °C) and the transition broadens, indicating thermal hysteresis associated with chain relaxation and enthalpy recovery during heating. Additional broad endothermic features in the range 115–155 °C correspond to hard-segment rearrangements and partial disruption of urethane-associated domains.

The PIR foam exhibits a markedly higher glass transition, with a *T_g_* midpoint at ~88 °C during heating and ~82 °C during cooling. The corresponding Δ*c_p_* (~0.40 J·g^−1^·K^−1^) is smaller than that of PUR, consistent with reduced segmental mobility arising from the rigid isocyanurate rings and polyester backbone. The higher *T_g_* values indicate that PIR remains in a glassy, dimensionally stable state over a broader temperature range. Elevated-temperature relaxation peaks occur at 135–175 °C, again at higher temperatures than for PUR, confirming the greater thermal robustness of the PIR network.

The DSC results presented in Table 4 thus show that PIR possesses significantly reduced soft-segment mobility, higher thermal transition temperatures, and a more constrained polymer network. These features directly correlate with the enhanced mechanical stability at elevated temperatures and the improved fire resistance observed in flammability testing.

In addition to the numerical values of the glass transition temperature, the characteristic shapes of the DSC curves provide important information on polymer network organization. The relatively sharp, step-like glass transition observed for the PUR foam during heating is indicative of higher soft-segment mobility and weaker topological constraints within the polyether-based network. In contrast, the broader and less pronounced glass transition step observed for the PIR foam reflects a more constrained network structure with restricted segmental motion, arising from the combined presence of isocyanurate crosslinks and aromatic polyester segments. The reduced thermal hysteresis between heating and cooling scans for PIR, compared with PUR, further indicates enhanced structural stability and limited chain relaxation during thermal cycling.

Both PUR and PIR heating curves exhibit broad endothermic features at temperatures significantly above the glass transition. These effects are not associated with melting phenomena, as rigid PUR and PIR foams form highly crosslinked, amorphous polymer networks. Instead, such endothermic events are commonly attributed to relaxation and partial reorganization of hard-segment-rich domains, including disruption of physical interactions such as hydrogen bonding within urethane- and isocyanurate-containing structures.

The combined TG–DTG and DSC results demonstrate a clear relationship between polymer architecture and thermal behavior: higher *T_g_* and lower Δ*c_p_* in PIR indicate a more rigid, highly crosslinked network, which provides:

- improved dimensional stability under thermal load,
- reduced creep and deformation near service temperatures, and greater resistance to pre-ignition softening in fire scenarios.

Higher degradation temperatures and increased char yield in PIR support improved fire performance, as the material

- resists structural collapse longer during heating,
- forms a stable, insulating carbonaceous skeleton, and releases combustible volatiles at slower rates.

Lower thermal stability and lower *T_g_* in PUR correlate with

- earlier softening and loss of mechanical stiffness above ~60 °C,
- negligible char formation,
- higher heat release rates and earlier peak HRR in cone calorimetry.

In summary, the thermal behavior of the two foams reflects their chemical structures: the PIR foam shows superior thermal stability, higher glass transition temperature, and enhanced char formation due to the presence of isocyanurate rings and aromatic polyester segments. These characteristics make PIR more suitable for high-temperature insulation and fire-sensitive applications compared to conventional PUR foam.

The results obtained in this study highlight several strengths and advantages of the proposed PIR systems formulated with recycled PET-based polyester polyols. In particular, the observed differences in thermal degradation behavior and glass transition temperature are consistent with trends reported for high-index PIR systems containing aromatic polyester polyols [13,15]. The combined presence of isocyanurate rings and aromatic segments promotes condensed-phase stabilization, delays the release of combustible volatiles, and enhances char formation, resulting in improved thermal resistance compared with conventional PUR foams. Importantly, the present results confirm that the incorporation of recycled PET-derived polyols does not compromise thermal stability, but instead contributes to increased network rigidity and structural integrity at elevated temperatures.

From an engineering perspective, these findings indicate that PET-based PIR foams can offer a favorable balance between enhanced fire performance and the use of recycled raw materials, without introducing adverse thermal trade-offs. Nevertheless, certain limitations and risks should be acknowledged. Only representative formulations were investigated, and the influence of formulation variability, processing conditions, and long-term aging effects was not addressed in the present study. Despite these limitations, the clear and systematic shift in degradation onset temperature and glass transition temperature suggests a robust structure–property relationship governing the thermal response of PIR systems. These results provide a solid foundation for further optimization of recycled PET-based PIR formulations targeted at high-performance and fire-resistant insulation applications.

### 3.2. Cone Calorimetry Analysis HRR

The heat release rate (HRR) curves obtained from cone calorimetry clearly distinguish the fire behavior of the PUR and PIR foams. The characteristic shapes, peak intensities, and times to peak directly reflect differences in their thermal decomposition pathways and condensed-phase stabilization mechanisms. The PUR curve displays a sharp, symmetrical peak with a maximum HRR of 350 kW/m^2^, occurring at approximately 400 s. This high and narrow peak indicates a fast-developing combustion process, typical for low-index polyurethane systems. Key parameters have been shown in Table 5. The key features are:

- rapid volatilization after ignition due to the cleavage of urethane linkages,
- minimal char formation, which allows heat and oxygen to easily penetrate the foam,
- gas-phase dominated burning, with the majority of combustible mass released within a short interval.

The enhanced fire performance of PIR foams results from a synergistic effect between thermally stable isocyanurate rings and aromatic PET-derived polyester segments. Isocyanurate structures delay backbone scission and reduce the rate of volatile fuel generation, while aromatic segments promote char formation and carbonization. Together, these mechanisms favor the formation of a cohesive condensed-phase barrier that limits heat and mass transfer during combustion.

The steep rise toward the peak shows that the production of flammable pyrolysis gases is rapid, while the equally steep decline after the peak suggests that the available fuel is exhausted quickly. This pattern is characteristic of PUR materials that lack condensed-phase stabilization and exhibit low thermal resistance.

The PIR curve exhibits a broader and much lower heat release profile, with a peak of 180 kW/m^2^ observed at 499 s. Two aspects of this behavior are technologically significant:

- the peak heat release rate (pHRR) is reduced by almost 50% compared with PUR,
- the time to peak HRR (t-pHRR) is delayed by nearly 100 s, indicating slower combustion kinetics.

During combustion, the synergistic effect between the isocyanurate network and the aromatic PET-based polyester segments becomes particularly evident in cone calorimetry. The isocyanurate-derived char provides structural integrity and thermal resistance, while the aromatic polyester fraction enhances char density and continuity. This combined effect results in the formation of a cohesive, thermally insulating surface layer that limits heat feedback from the flame, reduces oxygen diffusion, and suppresses the release of flammable volatiles. Consequently, the peak heat release rate is significantly reduced and the combustion process is prolonged but less intense.

This behavior is in good agreement with previously reported cone calorimetry studies on PIR foams, in which the formation of a coherent, thermally insulating char layer was identified as the dominant mechanism governing heat and mass transfer during combustion [11]. Importantly, the present results indicate that this mechanism is preserved in systems formulated with recycled PET-based polyester polyols.

This improvement is attributed to the formation of isocyanurate crosslinks and char-forming reactions, which:

- enhance thermal stability of the polymer matrix,
- reduce the rate of volatile fuel generation
- promote formation of a protective carbonaceous layer at the foam surface,
- limit heat and mass transfer between the flame and underlying material.

The broad shape of the PIR HRR curve indicates a controlled, less aggressive combustion process, where fuel is released steadily rather than explosively.

**Figure 8:**
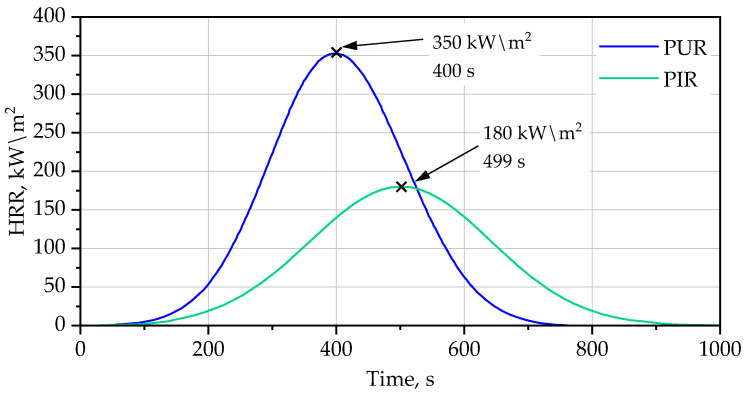
Cone calorimetry HRR curves for PUR and PIR foams.

Overall, the significant reduction in peak heat release rate and the delayed combustion behavior clearly demonstrate that PIR foams offer superior fire resistance, primarily due to their higher isocyanurate content and enhanced char-forming capabilities these capabilities are shown on Figure 8.

The consistency between delayed mass loss observed in TG–DTG analysis and reduced heat release measured by cone calorimetry confirms that the improved fire performance of PIR foams originates from condensed-phase stabilization driven by the synergistic interaction of isocyanurate structures and PET-derived aromatic segments.

### 3.3. Toxic Gas Emission

Commercial PUR foams typically contain halogen-based flame retardants, which contribute to the release of additional acidic and toxic gases (HCl, SO_2_) during combustion. According to PN-B-02855, materials with WLC50M values below 40 are classified as toxic, whereas values exceeding 40 correspond to moderately toxic materials. Based on this classification, the PIR foam exhibits a moderately toxic profile at selected decomposition temperatures, while falling into the toxic category at higher oxidative decomposition temperatures, which is typical for polymeric insulation materials. As shown in Table 6, standard PUR systems are typically classified as toxic, with WLC50SM values below 40. In contrast, the self-formulated PIR foam based on PET-derived polyol and halogen-free flame retardants shows significantly reduced emission of toxic gases and maintains a safer classification profile. This improvement can be attributed to absence of halogenated FR components, increased aromatic char formation (PET polyester + isocyanurate rings), reduced release of acidic decomposition products, slower decomposition kinetics (as confirmed by TG–DTG). The toxic gas emission analysis demonstrates that the PIR foam formulated with polyester polyol containing recycled PET exhibits lower toxic gas release and achieves a superior toxicological classification compared to standard construction-grade PUR foams, as shown in Table 6 and Table 7.

From a sustainability perspective, the incorporation of recycled PET-based polyester polyols enables partial substitution of virgin petrochemical raw materials and contributes to plastic waste valorization within a circular-economy framework. Importantly, the present results indicate that this sustainability-driven material strategy does not involve a trade-off in fire performance. On the contrary, the aromatic structure of PET-derived polyols supports char formation and condensed-phase stabilization, which directly contributes to reduced heat release and improved fire behavior. This observation highlights a beneficial synergy between environmental objectives and fire-safety requirements in the design of rigid PIR insulation materials.

## 4. Conclusions

This study provides a comparative assessment of the thermal behavior, fire performance, and toxic gas emission of rigid PUR and PIR foams formulated with recycled PET-based polyester polyols, with particular emphasis on the role of chemical structure in determining fire-related properties.

1. Rigid polyisocyanurate (PIR) foams formulated with recycled PET-based polyester polyols exhibit markedly higher thermal stability than conventional polyurethane (PUR) foams, as reflected by delayed thermal degradation and significantly increased char yield.
2. The combined presence of isocyanurate rings and aromatic polyester segments derived from PET promotes enhanced condensed-phase stabilization during thermal decomposition, resulting in reduced fire intensity and slower fire development.
3. PIR foams demonstrate higher glass transition temperatures and reduced segmental mobility compared with PUR foams, indicating improved dimensional stability and resistance to thermally induced softening under elevated temperature conditions.
4. Cone calorimetry results confirm that PIR foams are characterized by substantially lower peak heat release rates and delayed combustion dynamics, which is advantageous for insulation applications requiring enhanced fire safety performance.
5. Toxic gas emission analysis indicates that the halogen-free PIR formulation based on recycled PET polyols exhibits a more favorable toxicological profile compared with conventional PUR foams, particularly due to the absence of halogen-derived acidic gases and the reduced release rate of combustion products.
6. From a sustainability perspective, the use of recycled PET-based polyester polyols enables partial substitution of virgin petrochemical raw materials and contributes to plastic waste valorization within a circular-economy framework. Importantly, the present results demonstrate that this sustainability-driven material strategy does not compromise fire performance, but instead supports enhanced thermal stability and char formation, highlighting a positive synergy between environmental and fire-safety requirements.
7. Overall, the findings indicate that PIR foams based on recycled PET-derived polyester polyols represent a promising class of rigid insulation materials that combine improved fire performance, favorable toxicological characteristics, and sustainability-oriented material design.

## Figures and Tables

**Table 1:** Typical properties of PUR and PIR foams.

Property	Value
Density [kg/m^3^]	30–60
Glass transition temp. [°C]	120–160 PUR; 150–210 PIR
Thermal conductivity (λ) [W/m·K]	0.019–0.026
Process shrinkage [%]	0.3–3.0
Compressive strength [kPa]	120–250
Dimensional stability –30: +80 °C [%]	<1–3
Cell size [µm]	150–300
Continuous operating temperature [°C]	50.0–110
Industry application	wall insulation, roof insulation, refrigeration insulation, door insulation, sandwich panel core, automotive insulation

**Table 2:** Composition of rigid PUR foam formulation.

Component	Function	Content (pbw)
Polyether polyol (Rokopol^®^ G44)	Main polyol component	70.0
Polyester polyol (PET-based, HT 2000)	Aromatic modifier	30.0
n-Pentane	Physical blowing agent	14.0
TCPP (Tris(2-chloro-1-methylethyl) phosphate)	Halogen flame retardant	6.0
PMDETA (20% in polyol)	Urethane catalyst	0.30
DABCO^®^ TMR-2	Auxiliary amine catalyst	0.20
Potassium acetate (in glycol)	Trimerization promoter	0.05
Silicone surfactant	Cell stabilizer	1.50
Polymeric MDI (Voracor™ CD 345) index 110	Isocyanate component	calculated

**Table 3:** Composition of rigid PIR foam formulation.

Component	Function	Content (pbw)
Polyether polyol (Rokopol^®^ G44)	Secondary polyol	50.0
Polyester polyol (PET-based, HT 2000)	Main aromatic polyol	50.0
n-Pentane	Physical blowing agent	13.0
TEP (triethyl phosphate)	Halogen-free flame retardant	8.0
PMDETA (20% in polyol)	Urethane catalyst	0.20
DABCO^®^ TMR-2	Isocyanurate catalyst	0.40
Potassium acetate (in glycol)	Trimerization catalyst	0.30
Silicone surfactant	Cell stabilizer	1.50
Polymeric MDI (Voracor™ CD 345) index 300	Isocyanate component	calculated

**Table 4:** Key parameters of DSC curves.

System	Scan	*T_g_* Onset (°C)	*T_g_* Midpoint (°C)	*T_g_* Endset (°C)	Δ*c_p_* (J·g^−1^·K^−1^)	Higher-T Events (°C)	Qualitative Interpretation
PUR foam	heating	63.6	63.4	70.3	0.56	115, 155	Rigid PUR; *T_g_* just above service; moderate chain mobility; high-T relaxations start >100 °C
PUR foam	cooling	61.4	55.0	48.3	0.50–0.55	weak features >100	*T_g_* shifted lower (hysteresis, relaxation); glass transition still relatively low; confirms limited but noticeable segmental mobility
PIR foam	heating	82.0	88.0	95.0	0.40	135, 175	Highly crosslinked PIR; *T_g_* significantly higher; lower Δ*c_p_*; hard segments stable to higher T
PIR foam	cooling	90.0	82.0	74.0	0.35–0.40	weak events near 170–185	*T_g_* window shifted ~20–30 °C above PUR; small Δ*c_p_*; strong network rigidity and high thermal stability

**Table 5:** Cone Calorimetry parameters for PUR/PIR foam.

Parameter	PUR Foam	PIR Foam	Significance
pHRR	350 kW/m^2^	180 kW/m^2^	PIR reduces heat release by ~50%
t-pHRR	400 s	499 s	PIR delays combustion peak by ~100 s
Curve shape	sharp, narrow	broad, flattened	PIR burns slower and less intensely
Char effect	minimal	strong	PIR provides condensed-phase protection

**Table 6:** Toximetric indices for standard PUR foam based on polyether polyol (WLC50, WLC50M), *n* = 3.

Temperature [°C]	Toximetric Indices [g/m^3^] ^a^						WLC50 [g/m^3^]	WLC50M $\bar{x}$ [g/m^3^]
	CO	CO_2_	HCN	NO_2_	HCl	SO_2_		
450	34	142	1	0	1	55	55	36
550	159	408	11	0	1	9	—	
750	14	1097	1	0	3	45	45	

^a^ The values presented in the table represent mean values calculated from *n* measurements, where the number of measurements (*n*) complies with the requirements specified in the relevant standard.

**Table 7:** Toximetric indices for PIR foam based on recycled PET polyester polyol (WLC50, WLC50M), *n* = 3.

Temperature [°C]	Toximetric Indices [g/m^3^] ^b^						WLC50 [g/m^3^]	WLC50M $\bar{x}$ [g/m^3^]
	CO	CO_2_	HCN	NO_2_	HCl	SO_2_		
450	9	54	0	0	0	0	283	124
550	115	273	7	0	0	0	13	
750	10	914	0	0	0	2	76	

^b^ The values presented in the table represent mean values calculated from *n* measurements, where the number of measurements (*n*) complies with the requirements specified in the relevant standard.

## Data Availability

The original contributions presented in this study are included in the article. Further inquiries can be directed to the corresponding author.
